# Emergency Department Use among Patients with Mental Health Problems: Profiles, Correlates, and Outcomes

**DOI:** 10.3390/ijerph21070864

**Published:** 2024-06-30

**Authors:** Marie-Josée Fleury, Zhirong Cao, Guy Grenier

**Affiliations:** 1 Department of Psychiatry, McGill University, 1033, Pine Avenue West, Montreal, QC H3A 1A1, Canada; 2 Douglas Hospital Research Centre, Douglas Mental Health University Institute, 6875 LaSalle Blvd., Montreal, QC H4H 1R3, Canada; zhirong.cao@douglas.mcgill.ca (Z.C.); guy.grenier@douglas.mcgill.ca (G.G.)

**Keywords:** mental health problems, emergency department use, latent class analysis, profiles, adverse outcomes

## Abstract

Patients with mental health (MH) problems are known to use emergency departments (EDs) frequently. This study identified profiles of ED users and associated these profiles with patient characteristics and outpatient service use, and with subsequent adverse outcomes. A 5-year cohort of 11,682 ED users was investigated (2012–2017), using Quebec (Canada) administrative databases. ED user profiles were identified through latent class analysis, and multinomial logistic regression used to associate patients’ characteristics and their outpatient service use. Cox regressions were conducted to assess adverse outcomes 12 months after the last ED use. Four ED user profiles were identified: “Patients mostly using EDs for accessing MH services” (Profile 1, incident MDs); “Repeat ED users” (Profile 2); “High ED users” (Profile 3); “Very high and recurrent high ED users” (Profile 4). Profile 4 and 3 patients exhibited the highest ED use along with severe conditions yet received the most outpatient care. The risk of hospitalization and death was higher in these profiles. Their frequent ED use and adverse outcomes might stem from unmet needs and suboptimal care. Assertive community treatments and intensive case management could be recommended for Profiles 4 and 3, and more extensive team-based GP care for Profiles 2 and 1.

## 1. Introduction

Patients with mental health (MH) problems, including mental disorders (MDs), substance-related disorders (SRDs), and suicidal behaviors, are known to use emergency departments (EDs) frequently. A 2016 systematic review and meta-analysis [1] and a 2021 US study [2], respectively, found that 4% and 5.3% of ED visits were for MDs, including SRDs. A Canadian study found that in 2014–2015, 17% of ED users with MDs or SRDs had visited the ED at least four times for any reason, and that 22% of these patients had visited the ED on three to five consecutive years [3,4]. Some patients may thus be “recurrent” ED users who use EDs repeatedly over several years; some of them will be high or very high ED users, respectively, defined as 3+ [5,6] or 8+ [7,8] ED visits/year. Patients may use the ED for severe conditions such as suicidal behaviors, or for milder ones treatable in outpatient care [9]. Some use the ED in a more seasonal manner, during periods where they may be having a harder time—e.g., the holiday season [10]. The ED may also be the entry point to care for patients with incident MDs (new cases), which according to some studies could represent as many as 45% of all patients using the ED [3,11]. While EDs provide rapid access to acute MH care [12], limited access to outpatient care or receiving inadequate care may also lead to frequent ED use [13], thus contributing to ED overcrowding [14], higher healthcare costs [15], and adverse outcomes (e.g., hospitalization, death) [16]. ED users with MH problems are a heterogeneous population. Using person-centered approaches like latent class analysis, which correlates specific user characteristics rather than variables on heterogeneous populations, may present solutions for finding distinct profiles among ED users [17]. Improving our understanding of ED user profiles by associating them with patient characteristics, service use and subsequent adverse outcomes could help recommend care that is better adapted to these patients’ needs.

Several studies assess profiles of patients who use the ED frequently for any medical reasons, identifying four [18,19,20,21,22] or five [23,24] high ED user profiles, with one [20,22] to four [23,24] profiles mainly comprising patients with MDs or SRDs. None of these studies link ED user profiles to subsequent health outcomes. Few studies identify ED user profiles exclusively among patients with MDs [25,26,27]. One study found four profiles; the profile with the largest number of high ED users also reported the lowest quality of life [25]. Another study on patients with incident MDs identified five profiles: the profile with the most complex and serious health conditions included more high ED users, had the lowest continuity of care from general practitioners (GPs) and psychiatrists, and the worst adverse outcomes [26]. Another study investigating high ED users with MDs identified three profiles mainly made up of 3-year recurrent very high ED users and 2-year recurrent high ED users with poor health conditions and high risk of death [27]. Recurrent high and very high ED users were reported to be mostly men [21] of lower socioeconomic status [28] with worse health and social conditions; most had a severe MD [27], were high users of health services in general, but did not necessarily receive adequate care [27]. These characteristics are usually associated with a higher risk of hospitalization and premature death [29,30]. 

To our knowledge, no prior study has assessed ED user profiles by considering patients with various MH problems (MDs, SRDs, and suicidal behaviors), whether incident cases or not, and by investigating the intensity, recurrence, triage priority, and dispersion of ED use over a 4-year period. This study is original in that it also examined outpatient service use over the 12 months before the last ED use to assess whether more continuous, intensive, diversified, and regular outpatient service use could be associated with ED user profiles and subsequent risks of hospitalization and premature death. Most previous studies have used hospital administrative records instead of provincial databases that would include broader information on outpatient care use. Such information could contribute to our understanding of ED use profiles. Based on provincial databases, this longitudinal study thus aimed to identify profiles of ED users with MH problems, to associate these profiles with patient sociodemographic and clinical characteristics and service use, and to subsequently link them to adverse outcomes.

## 2. Materials and Methods

### 2.1. Study Design, Cohort, and Data Sources 

This study investigated a 2012–2017 cohort of 11,938 patients with MH problems who had used one of six psychiatric EDs in Quebec (Canada). To be eligible, patients had to be enrolled in Quebec Health Insurance Plan, aged 12 or older, and have used an ED between 1 April 2012 and 31 March 2016. Patients hospitalized for over 120 days in the year before their last ED visit were excluded, to ensure accurate assessment of outpatient care. Patients were identified by their name and their medical insurance number from the Quebec Health Insurance Plan. The Quebec Health Insurance Plan databases used for this study included the health insurance registry (FIPA, containing patient sociodemographic information, i.e., sex, birth date, and age), the physician claims database (RAMQ), the ED use database (BDCU), the hospital inpatient database (MED-ECHO), the community healthcare center database (I-CLSC, mostly psychosocial services), and the vital statistics death database (RED). Variables that appeared in several databases were merged (see Table 1 footnotes). Diagnostic codes were classified according to the *International Classification of Diseases*, ninth or tenth revisions (Appendix A). This study followed the Strobe guideline for epidemiological studies [31]. The ethics committee of the Douglas Mental Health University Institute approved the study protocol (IUSMD-20-26).

### 2.2. Study Variables

The variables used to formulate patient profiles included: ED use for incident MDs or using the ED as a primary MH service (measured from 1 April 2014 to 31 March 2015); number of years of ED use; mean number of ED visits/year; very high ED use; recurrent high ED use; percentage of ED visits with high triage priority; low ED use dispersion. All these were monitored from 1 April 2012 to 31 March 2016. As in previous research, incident MDs were defined as diagnoses made after a 2-year period without any MD, SRD, or MH consultation [11]. Very high ED use was defined as 8+ visits/year [7], high ED use as 3+ visits/year [5], and recurrent high ED use meant these visits occurred over 2+ consecutive years [32]—all minimum benchmarks. In agreement with the Canadian Triage Acuity Scale, ED triage priority assessed illness acuity on a scale of 1 to 5, with level 1 representing the most urgent cases and levels 4 and 5 deemed suitable for outpatient care [33]. The proportion of high-priority visits (levels 1–3) was calculated out of all ED visits (levels 1–5). Dispersion was used to measure ED use over the year, with patterns that could occur over a short period, such as during the holidays [21]. Having 2+ ED visits within a 90-day period over 2+ consecutive years was defined as “low dispersion” [34].

Sociodemographic correlates included sex at birth, age group, residing in more materially or socially deprived areas, all recorded at the initial ED use; violent/disturbed behaviors or social problems were documented from the first to the last ED use. Based on the smallest geographic areas outlined in recent versions of the Canadian census, Material and Social Deprivation Indexes were categorized into two groups: the least and moderately deprived areas (quintiles 1–3); the more deprived areas (quintiles 4–5, including areas not assigned [e.g., homelessness]). The Material Deprivation Index gauged employment rates, average income, and the proportion of residents without a high school diploma, while the Social Deprivation Index assessed the proportion of residents living alone, without a spouse, and single-parent families [35]. Violent/disturbed behaviors and social problems (e.g., homelessness) were identified by ED triage nurses. 

Clinical correlates were assessed between the first and last ED use and included the principal MD, SRDs (alcohol/drug use, induced disorders, intoxication, withdrawal), suicidal behaviors (suicide ideation/attempt), chronic physical illnesses (e.g., cardiovascular illnesses) and their severity, and ambulance/stretcher use. The principal MD were categorized as a serious MD (schizophrenia spectrum and other psychotic disorders, bipolar disorders), personality disorders, or a common MD (depression, anxiety, adjustment, attention deficit/hyperactivity disorders). Chronic physical illnesses were assigned a severity score ranging from 0 to 3 adapted from the Elixhauser and Charlson Comorbidity Indexes, with higher scores indicating a higher risk of death [36]. The Charlson Comorbidity Index measures 17 medical conditions [37]; the Elixhauser measures 30. Together, they cover 32 chronic physical illnesses [36]. Being transported to an ED by ambulance or using a stretcher served as proxies for the severity of conditions, calculated by dividing the number of times these measures were utilized by the patient’s total number of ED visits. 

Measured in the year prior to the last ED visit, service use correlates included having a usual GP or psychiatrist, high continuity of physician care, frequency of MH outpatient consultations by any physician, psychosocial interventions from community healthcare centers, and high regularity of outpatient care. A proxy for family physician, “usual GP” required a minimum of two consultations with the same GP or with a GP in a family medicine group, which is a medical clinic where GPs work in groups that include clinicians, mostly nurses and social workers; patients are also registered in these clinics, which offer comprehensive patient care [38]. “Usual psychiatrist” required 2+ outpatient consultations with the same psychiatrist, or one psychiatric consultation showing collaborative care with the usual GP [39]. Continuity of physician care was gauged using the Usual Provider Continuity Index [40], which calculates the proportion of consultations with the usual physicians relative to all physicians consulted—a score of ≥0.80 indicates high continuity of care [41]. The frequency of outpatient consultations measured care intensity, 4+ consultations/year being the minimum benchmark [42,43]. In Quebec, public psychosocial services are mainly provided by community healthcare centers [44]. High regularity of outpatient care integrated care received from usual physicians and psychosocial services, requiring at least one patient–clinician contact in each of the four 3-month periods of the year, which indicated close patient follow-up care over time [45]. 

Adverse outcomes were monitored for 12 months after the last ED use (2012 to 2016) and included hospitalization for MH reasons and death for any cause. These metrics are key indicators for measuring adverse outcomes utilizing health administrative databases [46,47,48]. 

### 2.3. Data Analysis

Considering that missing values were less than 1%, a complete case analysis was used [49]. Univariate analyses included frequency distributions for categorical variables. Latent class analysis (LCA) [50,51] was used to identify patient profiles based on ED use variables. Compared to standard cluster analysis, LCA provides a stronger alternative for assessing model fit and captures classification uncertainty more effectively [52]. A series of increasingly complex models (adding classes) was evaluated to determine the optimal number of latent classes. Following standard practice, the Bayesian information criterion (BIC) [53] and the entropy value [54] were calculated to identify the best model. Comparative analyses of correlates were conducted across patient profiles using bivariate multinomial logistic regression, based on the patient sociodemographic and clinical characteristics, and service use. Relationships between patient profiles and adverse outcomes were tested using Cox regressions, adjusted for age and sex. Unadjusted risk ratios and adjusted hazard ratios were calculated. LCA was performed with SAS 9.4 [55] and other analyses were carried out with Stata 17 [56]. 

## 3. Results

### 3.1. Cohort Description

Of the initial 11,938 patients, 256 were excluded because they had been hospitalized >120 days in the 12 months before their last ED use. In the final cohort of 11,682 patients, 24% had incident MDs, 20% had used EDs over the 4-year period, 25% had 4+ ED visits/year, 13% were very high ED users, 30% had high triage priority in 67–100% of ED visits, and 37% showed low ED use dispersion (Table 1). Half the patients (51%) were women; 55% were 30–64 years old and also lived in more materially or socially deprived areas. About half had a serious MD, 40% had SRDs, and 44% exhibited suicidal behaviors or chronic physical illnesses. In the 12 months before the last ED use, 50% had a usual GP, 39% a usual psychiatrist, 61% showed high continuity of physician care, 40% and 53% did not receive care from any outpatient physician or community healthcare center, respectively, and 37% received a high regularity of outpatient care. In the 12 months following the last ED use, 10% were hospitalized and 2% died.

**Table 1:** Characteristics of patients with mental health (MH) problems using emergency departments (EDs) (*n* = 11,682).

		*n*	%
ED use variables (measured from 1 April 2012 to 31 March 2016, or other as specified)			
ED use for incident mental disorders (MDs) or patients who use EDs as their first MH service (measured form 1 April 2014 to 31 March 2015)		2808	24.04
Years of ED use ^a,b^	1	2686	22.99
	2	3550	30.39
	3	3052	26.13
	4	2394	20.49
Mean ED visits per year ^a,b^	1	2902	24.84
	2	3762	32.20
	3	2142	18.34
	4+	2876	24.62
Very high ED use (8+ visits/year) ^a,b^		1490	12.75
Recurrent high ED use (3+ visits/year) for at least 2 consecutive years ^a,b^		2438	20.87
Percentage of ED visits with high triage priority (1–3/1–5) ^a^	0–32%	4359	37.31
	33–66%	3871	33.14
	67–100%	3452	29.55
Low dispersion of ED use (2+ visits within 90 days for at least 2 years) ^a,b^		4362	37.34
Sociodemographic correlates (measured at the first ED use, or other as specified)			
Sex ^c^	Women	6003	51.39
Age (years) ^c^	12–29	4002	34.26
	30–64	6466	55.35
	65+	1214	10.39
Living in more materially and socially deprived areas: indexes 4–5 or areas not assigned ^b^		6470	55.38
Violent/disturbed behaviors or social problems (measured from the first to the last ED use) ^a^		3534	30.25
Clinical correlates (measured from the first to the last ED use, or other as specified)			
Principal MD ^a,b,d^	None	925	7.92
	Serious MD	5613	48.05
	Personality disorders	1653	14.15
	Common MD	3491	29.88
Substance-related disorders (SRDs) ^a,b,d^		4684	40.10
Suicidal behaviors (suicide ideation/attempt) ^a,b,d^		5092	43.59
Chronic physical illnesses ^a,b,d^		5178	44.32
Severity of chronic physical illnesses (a 3+ score) ^a,b,d^		1580	13.53
Ambulance/stretcher use ≥50% ^a^		2793	23.91
Service use correlates (measured for the 12 months before the last ED visit)			
Usual general practitioner (GP) ^b,e^		5888	50.40
Usual psychiatrist ^b^		4499	38.51
High continuity of physician care (usual GP and outpatient psychiatrist) ≥0.80 ^b,e^		7145	61.16
Frequency of MH outpatient consultations from any physician ^b,e^	0	4704	40.27
	1–3	3735	31.97
	4+	3243	27.76
Psychosocial interventions from community healthcare centers ^e^	0	6145	52.60
	1–3	2476	21.20
	4+	3061	26.20
High regularity of outpatient care (including usual GP, usual psychiatrist, and psychosocial clinicians from community healthcare centers) within the four 3-month periods of the year ^b,e^		4371	37.42
Outcome (measured for the 12 months after the last ED visit)			
Hospitalization for MH reasons ^d^		1159	9.92
Death for any cause ^f^		214	1.83

^a^ Banque de données communes des urgences (BDCU—ED Database); ^b^ Régie de l’assurance maladie du Québec (RAMQ—Physician Claims Database); ^c^ Fichier d’inscription des personnes assurées (FIPA, Health Insurance Registry); ^d^ Maintenance et exploitation des données pour l’étude de la clientèle hospitalière (MED-ECHO—Hospital Inpatient and Day Surgery Database); ^e^ Système d’information permettant la gestion de l’information clinique et administrative dans le domaine de la santé et des services sociaux (I-CLSC—Community Healthcare Center Database); ^f^ Fichier des décès du registre des évènements démographiques (RED, Vital Statistics Death Database).

### 3.2. Patient Profiles

The four-class solution was selected based on the smallest BIC (three-class = 3293; four-class = 2361; five-class = 2368), with an entropy value of 0.84–˃0.8 indicating a limited class overlap. Accounting for 30% of the sample, Profile 1 included the most patients with incident MDs (47%) or who use an ED as their first MH service (Table 2). Most Profile 1 patients (83%) had a mean of one ED visit/year, with 44% using an ED only once in the four years considered, 27% using it over 2 years, and 25% over 3 years. Profile 1 included the most patients (45%) with high triage priority on 67–100% of ED visits. None were recurrent high or very high ED users. Profile 1 was labelled “Patients mostly using EDs for accessing MH services”. 

Profile 2 (32% of sample) included the most patients with 2 years of ED use and a mean of 2 ED visits/year. It had the least number of patients with high triage priority on 67–100% of ED visits and included no recurrent high ED users—though 4% were very high ED users. Profile 2 was labelled “Repeat ED users”.

Profile 3 (19% of sample) included the most patients with 3 years of ED use and a mean of 3 ED visits/year. In this profile, 29% were recurrent high ED users, but none were very high ED users. Almost all Profile 3 patients had 2+ visits within 90 days over at least 2 years and showed low ED use dispersion. Profile 3 was labelled “High ED users”.

Profile 4 (19% of sample) comprised the most patients with 4 years of ED use. Almost all of them averaged 4+ ED visits/year, with low ED use dispersion. Most (82%) were recurrent high ED users, and 61% very high ED users. Profile 4 was labelled: “Very high and recurrent high ED users”.

### 3.3. Patient Profiles and Associated Sociodemographic and Clinical Characteristics and Service Use

Compared to Profile 1, Profiles 4, 3, and 2 patients were 22%, 18%, and 12% more likely to be women, and 83%, 70%, and 21% more likely to be 65+ years old, respectively. Profiles 4 and 3 patients were also 42% and 25% more likely to be 30–64 years old compared to those in Profile 1. Profiles 4, 3, and 2 patients were 89%, 47%, and 29% more likely to live in materially and socially deprived areas and had five or three times and 88% higher risk of exhibiting violent/disturbed behaviors or social problems, respectively, than those of Profile 1. 

Compared to Profile 1, Profiles 4, 3, and 2 had 30, 9, and 3 times more risk of having a serious MD as principal MD; 12, 5, and 2 times more risk of personality disorders; 3 or 1 times and 85% to have a common MD; and 4 or 2 times and 36% SRDs, respectively. Compared to Profile 1, the risk of suicidal behaviors for Profiles 4, 3, and 2 was 1 time, 40% and 14% higher, and the likelihood of chronic physical illnesses with a 3+ severity score being 2 or 1 times and 14% higher, respectively. And compared to Profile 1, Profiles 4, 3, and 2 patients had an 81%, 80%, and 38% lower risk of being transported to EDs by ambulance or on a stretcher at least 50% of the time, respectively. 

Profiles 2, 3, and 4 patients were 28%, 17%, and 14% more likely to have a usual GP, and were 1, 2, and 4 times more likely to have a usual psychiatrist, respectively. Profiles 2 and 3 patients were 17% and 16% more likely to have a high continuity of physician care compared to those of Profile 1. Profiles 4, 3, and 2 patients had 94%, 71%, and 58% more chances of receiving at least four MH outpatient consultations from any physician, and were four and two times, and 73% more likely to receive at least four interventions from community health centers, respectively, than those of Profile 1. Profiles 4 and 3 patients were also four and two times more likely than those in Profile 1 to receive a high regularity of outpatient care (Table 3). 

### 3.4. Associations between Patient Profiles and Adverse Outcomes

Compared to Profile 1, risks of hospitalization for MH reasons were five times higher in Profile 4, three times higher in Profile 3, and 74% higher in Profile 2. Risk of death for any cause was higher in Profiles 4 and 3 (96%, 66%) than in Profile 1 (Table 4). 

## 4. Discussion

This study examined patients with MH problems over a 4-year period in order to identify ED user profiles, link correlates to these profiles, and assess subsequent risk of hospitalization and death. As previous ED studies have shown [57,58], patients in this study were quite vulnerable overall: about half were poor or isolated, affected by serious MDs, SRDs, chronic physical illnesses, or suicidal behaviors. One quarter of patients had a mean of 4+ ED visits in the 4-year ED follow-up period. This number is higher than that found in previous studies [59,60], which could be because patients were only recruited in large psychiatric EDs and had conditions such as suicidal behaviors. About half did not receive any physician or psychosocial service in the year before their last ED use, and only a third received a high regularity of care. The percentage of first ED use for incident MDs found here (24%) was lower than in other studies (±50%) [3,11], which could also be due to patients only assessed in psychiatric EDs. Four profiles of ED users with MH problems were identified, each substantially different from the others. 

Accounting for one-third of the cohort, Profile 1 patients differed from other profiles as they used EDs the least during the 4 years considered. About half of them had entered the MH system through EDs and had not received MD or MH care during the 2 previous years; 83% showed a mean of only one ED visit. Roughly half showed only 1 year of ED use, about a quarter had 2 years, and the other quarter, 3 years. Profile 1 ED use was deemed the most urgent by the triage nurses, probably because they lacked MH care and had waited to have acute symptoms before going to the ED. The fact this profile included more men and younger patients may also explain these care-seeking behaviors: compared to women and older patients, men and younger patients are known to use less MH services, and only as their last resort [61,62]. Profile 1 patients also showed lower risk factors: they were not as poor and isolated and had fewer complex conditions than Profiles 4 and 3, especially in terms of serious MDs and SRDs. These characteristics explain why, of all profiles, Profile 1 patients also received the least MH care within the year of their last ED use. Fewer of them received care from psychiatrists and psychosocial services, and they had the lowest regularity of care. Their low ED use and MH conditions explain why they showed the lowest risks of hospitalization and death. Profile 1 patients may be easily treated in primary care [63], may benefit from extended team-based GP care (nurses, social workers) mirroring the chronic care model [64], including collaborative care with psychiatrists [65,66]. Brief intervention ED teams (e.g., ED liaison agents, ED case management) [67,68] might also be beneficial as they would provide care to these patients before they could be transferred to adequate outpatient care. 

Accounting for about one-fifth of the cohort, Profile 4 contrasted from Profile 1 as its patients had the worst ED use, poor social and material conditions, violent/disturbed behaviors, and were most affected by serious MDs, SRDs, suicidal behaviors, and severe chronic physical illnesses, all of which are characteristics of very high and recurrent high ED use [27,58,69,70]. The fact this profile featured very high ED users may explain why about half of them showed high triage priority on 33 to 66% of ED visits. They received the least GP care after Profile 1, but even though they were high ED users, they received the most psychiatrist and psychosocial care—high ED users are known to also be high MH care users [71,72]. Patients with these conditions are usually more likely to be followed in psychiatry rather than primary care, since GPs are often uncomfortable treating patients with these severe conditions [73,74]. Profile 4 patients received more outpatient care but may still have elevated unmet needs, as their continuity of care did not differ from that of Profile 1. These characteristics explain why Profile 4 showed the highest risk of hospitalization and death—hospitalizations are known to be frequent among patients affected by serious MDs and chronic physical illnesses [75]. Perhaps Profile 4 patients also had a higher risk of death because they were older and had severe chronic physical illnesses and suicidal behaviors—the latter being also associated with MDs and SRDs [76]. These patients may be flagged in ED medical records, so information about their high ED use can be made readily available to ED clinical teams to make sure they receive appropriate intensive care and have an individual care plan. ED staff could also work closely with outpatient clinicians to ensure these patients receive adequate care for their serious conditions. Assertive community treatments integrating SRD treatments [77] might also be suggested for very high and recurrent high ED users such as those in Profile 4. 

Encompassing almost 20% of the cohort, Profile 3 resembled Profile 4, but with milder health-seeking behaviors and risk factors. These patients were mostly high ED users, with about a third using EDs recurrently over 3 consecutive years. Like those of Profile 4, they were regular ED users, with 2+ ED uses in each 3-month period over 2+ years. Compared to other profiles, they were transported more often to EDs by ambulance and/or on a stretcher. It is possible that Profile 3 included more involuntary ED visits than Profiles 1 and 2, as patients with serious MDs and co-occurring disorders, violent/disturbed behaviors or social problems, and who are isolated and older are more prone to involuntarily solicit acute care [78]. Risks of hospitalization and death were lower in Profile 3 than in Profile 4, but higher than in the two other profiles. Intensive case management [79,80] and integrated MD-SRD treatments [77] may be recommended for Profile 3 patients. Intervention plans [81] may also be developed in the ED in close collaboration with outpatient teams to better target these patients and reduce their high ED use.

Accounting for 32% of the cohort, Profile 2 patients were characterized by repeated ED use, nearly half of them having 2 years of ED use. High triage priority was the lowest of all profiles: only 20% showed high priority 67–100% of the time. This means that, in most cases, these patients could have avoided using the ED. Profiles 2 and 1 had almost identical percentages in each age group and showed similar regularity of outpatient care, though Profile 2 patients had worse conditions and received more outpatient care than Profile 1—but less than Profiles 4 and 3. More Profile 2 patients had a usual GP than those of Profiles 3 and 4, perhaps because they had less serious MDs and co-occurring disorders [82]. These conditions give Profile 2 a higher risk of hospitalization compared to Profile 1, though risk of death was similar in both. Like those of Profile 1, Profile 2 patients may benefit from extended team-based GP care coupled with more psychosocial services, including help for crisis resolution [83] and peer support, as these may prevent the types of ED use nurses often perceive as non-urgent. Access to brief intervention teams providing follow-up care after ED use [68,84], transitioning patients to outpatient care, short-stay crisis units [85], and home-based treatments [86] might also be appropriate for these patients to avoid acute care. 

This study has limitations. First, the study database did not include information on issues linked to ED use like psychotic crises or high levels of stress, on programs such as assertive community treatment or intensive case management, on services received from private sector psychologists and addiction treatment centers, or on community-based services (e.g., crisis centers). Information on these services would have improved our understanding of outpatient care use and of its impact on ED use. However, even as these services are key in responding to patient needs, they remain mostly underfunded in Quebec and generally offer limited services to patients like those in this cohort [44,87,88]. Second, this study only measured hospitalization and death as adverse outcomes because other outcomes are unfortunately not documented in health administrative databases. Third, patients with long periods of hospitalization before their last ED use were excluded from this study, but they only comprised 2% of the sample. Fourth, materially or socially deprived conditions were based solely on the area where the patient lived. Some patients may live in poor areas but show a high household income, others may live alone or be single parents and have a strong social network, but unfortunately the study database did not contain information on the patients’ household income or social network. Fifth, it was hard to compare the profiles identified in this study with those found in the previous literature, as research is scarce in this area, and rarely considers similar variables. Finally, the findings may not be generalizable beyond psychiatric EDs, large urban settings, or contexts lacking universal health coverage for vulnerable populations.

## 5. Conclusions

This study identified four ED use profiles among patients with MH problems. About a quarter of patients showed a mean of 4+ ED visits in the four consecutive years that were considered. These patients were quite vulnerable and needed more adequate care to respond to their substantial needs. Profiles 4 and 3 had the most ED use, the most severe conditions and received the most outpatient care, demonstrating the fairness in accessibility of Quebec’s public health system, which prioritizes the most vulnerable populations. The risk of hospitalization and death was also higher in these profiles, especially when compared to Profile 1 patients, who used EDs less frequently and primarily to access MH care. The frequent ED use and adverse outcomes of Profiles 4 and 3 might stem from unmet needs and suboptimal care. Based on this study’s findings, more intensive interventions are recommended for Profiles 4 and 3, including integrated MD-SRD treatments, assertive community treatments, and intensive case management. Profiles 2 and 1 could benefit from extended team-based GP care and crisis resolution teams. Strategies like intervention plans, short-stay crisis units, and home-based treatments should be more widely deployed in EDs to reduce frequent and recurrent ED use. Overall, patients should be more accurately identified in the ED, and integrated work should be carried out with outpatient services. Future research should favor mixed methods to investigate the types of outpatient care that are not considered in administrative databases and evaluate their impact on ED use. Understanding the perceived unmet needs that can lead to ED use may also be important to improve patient care and reduce ED use. 

## Figures and Tables

**Table 2:** Patient profiles based on their emergency department (ED) use (*n* = 11,682).

		Profile 1: Patients Mostly Using EDs for Accessing Mental Health (MH) Services	Profile 2: Repeat ED Users	Profile 3: High ED Users	Profile 4: Very High and Recurrent High ED Users
		%	%	%	%
Profile size (measured from 1 April 2012 to 31 March 2016, or other as specified)		29.93	32.20	19.36	18.50
ED use for incident mental disorders (MDs) or patients who use EDs as their first MH service (measured from 1 April 2014 to 31 March 2015)		46.61	17.86	15.87	6.80
Years of ED use	1	44.09	30.41	0.00	0.00
	2	27.08	47.93	19.32	16.80
	3	25.14	14.33	45.09	28.41
	4	3.69	7.34	35.59	54.79
Mean ED visits per year	1	82.99	0.00	0.00	0.00
	2	14.90	63.05	38.42	0.00
	3	2.12	22.04	54.33	0.46
	4+	0.00	14.91	7.25	99.54
Very high ED use (8+ visits/year)		0.00	4.70	0.00	60.76
Recurrent high ED use (3+ visits/year) for at least 2 consecutive years		0.00	0.00	29.13	82.32
Percentage of ED visits with high triage priority (1–3/1–5)	0–32%	40.69	40.11	35.99	28.37
	33–66%	14.36	39.98	36.38	48.22
	67–100%	44.95	19.91	27.63	23.42
Low dispersion of ED use (2+ visits within 90 days over at least 2 years)		0.00	0.00	98.41	98.84

**Table 3:** Associations between patient profiles based on their emergency department (ED) use, sociodemographic and clinical characteristics, and service use, using multinomial regression (*n* = 11,682) (Reference: Profile 1).

		Profile 1: Patients Mostly Using EDs for Accessing Mental Health (MH) Services		Profile 2: Repeat ED Users		Profile 3: High ED Users		Profile 4: Very High and Recurrent High ED Users	
		%	RR	%	RR	%	RR	%	RR
Profile size		29.93		32.20		19.36		18.50	
Sociodemographic correlates (measured at the first ED use, or other as specified)									
Women		48.78	---	51.54	1.12 *	52.87	1.18 *	53.77	1.22 *
Age (years)	12–29	37.58	---	35.94	---	31.17	---	29.2	---
	30–64	53.85	---	54.09	1.05	55.84	1.25 *	59.46	1.42 *
	65+	8.58	---	9.97	1.21 *	13.00	1.83 *	11.34	1.70 *
Living in more materially and socially deprived areas: indexes 4–5 or areas not assigned		48.64	---	54.94	1.29 *	58.18	1.47 *	64.14	1.89 *
Violent/disturbed behaviors or social problems (measured from the first to the last ED use)		16.21	---	26.69	1.88 *	34.08	2.67 *	55.16	6.36 *
Clinical variables (measured from the first to the last ED use)									
Principal MD	None	16.27	---	6.54	---	3.58	---	1.34	---
	Serious MD	27.94	---	47.08	4.19 *	58.00	9.43 *	71.86	31.19 *
	Personality disorders	12.87	---	14.46	2.80 *	15.74	5.56 *	14.02	13.21 *
	Common MD	42.92	---	31.92	1.85 *	22.68	2.40 *	12.77	3.61 *
Substance-related disorders (SRDs)		26.59	---	32.93	1.36 *	49.91	2.75 *	64.14	4.94 *
Suicidal behaviors (suicide ideation/attempt)		37.06	---	40.24	1.14 *	45.14	1.40 *	58.35	2.38 *
Severity of chronic physical illnesses (a 3+ score)		9.06	---	10.21	1.14 *	16.67	2.01 *	23.23	3.04 *
Ambulance/stretcher use ≥50%		37.35	---	27.03	0.62 *	10.88	0.20 *	10.37	0.19 *
Service use correlates (measured for the 12 months before last ED use)									
Usual general practitioner (GP)		47.07	---	53.16	1.28 *	51.02	1.17 *	50.35	1.14 *
Usual psychiatrist		21.16	---	38.01	2.28 *	47.04	3.31 *	58.54	5.26 *
High continuity of physician care (usual GP and outpatient psychiatrist) ≥0.80		58.94	---	62.71	1.17 *	62.51	1.16 *	60.67	1.07
Frequency of MH outpatient consultations from any physician	0	53.99	---	38.22	---	34.57	---	27.58	---
	1–3	30.28	---	33.97	1.58 *	33.16	1.71 *	29.99	1.94 *
	4+	15.73	---	27.80	2.50 *	32.27	3.20 *	42.43	5.28 *
Psychosocial interventions from community healthcare centers	0	65.77	---	54.23	---	46.46	---	34.89	---
	1–3	17.62	---	22.14	1.52 *	22.68	1.82 *	23.79	2.55 *
	4+	16.61	---	23.63	1.73 *	30.86	2.63 *	41.32	4.69 *
High regularity of outpatient care (including usual GP, usual psychiatrist, and psychosocial clinicians from community healthcare centers) within the four 3-month periods of the year		22.16	---	35.09	1.90	45.31	2.91 *	57.89	4.83 *

* *p*-value < 0.05. RR = risk ratio from bivariate multinomial regression.

**Table 4:** Associations between patient profiles based on their emergency department (ED) use and adverse outcomes, using Cox model (*n* = 11,682) (measured within 12 months after the last ED visit during the 2012–2016 period) (Reference: Profile 1).

	Profile 1: Patients Mostly Using EDs for Accessing Mental Health (MH) Services		Profile 2: Repeat ED Users		Profile 3: High ED Users		Profile 4: Very High and Recurrent High ED Users	
	%	HR	%	HR	%	HR	%	HR
Profile size	29.93		32.20		19.36		18.50	
Hospitalization for MH reasons	4.06	---	6.67	1.74 *	11.14	2.87 *	23.79	5.77 *
Death for any cause	1.14	---	1.41	1.09	2.61	1.66 *	2.87	1.96 *

* *p*-value < 0.05. HR = Hazard ratio. Each HR was adjusted for age and sex.

## Data Availability

Restrictions apply to the availability of these data. Data were obtained from the Quebec CAI and are available from the authors with the permission of the CAI.

## Appendix A

Codes for mental disorders (MDs), substance-related disorders (SRDs), suicide attempts, physical illnesses, and cause of death according to the *International Classification of Diseases*, Ninth and Tenth revisions.

**Diagnoses**	**International Classification of Diseases, Ninth Revision (ICD-9)**	**International Classification of Diseases, Tenth Revision, Canada (ICD-10-CA)**
MDs ^a^ Serious MDs		
Schizophrenia spectrum and other psychotic disorders	295 * (schizophrenic disorders); 297 * (paranoid states); 298 * (other nonorganic psychoses)	F20 * (schizophrenic disorders); F22 * (persistent delusional disorders); F23 (acute and transient psychotic disorders); F24 * (induced delusional disorder); F25 * (schizoaffective disorders); F28 * (other psychotic disorder not due to a substance or known physiological condition); F29 * (unspecified psychosis not due to a substance or known physiological condition); F448 (other dissociative and conversion disorders); F481 (depersonalization-derealization syndrome)
Bipolar disorders	2960–2966 (manic disorders); 2968 (other affective psychoses); 2969 (unspecified affective psychoses)	F300–F302, F308, F309 (manic episode); F310–F317, F318, 319 (bipolar episode)
Personality disorders	3010 (paranoid personality disorder); 3011 (affective personality disorder); 3012 (schizoid disorder); 3013, 3014 (obsessive-compulsive personality disorder); 3015 (histrionic personality disorder); 3016 (dependent personality disorder); 3017 (antisocial personality disorder); 3018 (other personality disorders); 3019 (unspecified personality disorder)	F600 (paranoid personality disorder); F61 (mixed and other personality disorders); F340 (cyclothymic disorder); F341 (dysthymic disorder); F601 (schizoid personality); F603 (borderline personality disorder); F605 (obsessive-compulsive personality disorder); F604 (histrionic personality disorder); F607 (dependent personality disorder); F602 (antisocial personality disorder); F609 (unspecified personality disorder); F21 (schizotypal personality); F606 (avoidant personality disorder); F608 (other specified personality disorders); F681 (factitious disorder); F688 (other specified disorders of adult personality and behaviour); F69 (unspecified disorder of adult personality and behaviour)
Common MDs		
Depressive disorders	3004 (neurotic depression) *; 311, 3119 * (depressive disorder, not elsewhere classified)	F320–F323 (major depressive disorder, single episode); F328 (other depressive episodes); F329 (depressive episode, unspecified); F330–F334 (major depressive disorder, recurrent); F338 (other recurrent depressive disorders); F339 (recurrent depressive disorder, unspecified); F348 (other persistent mood [affective] disorders); F380, F381 (persistent mood [affective] disorder, unspecified); F388 (other specified mood [affective] disorders); F39 (unspecified mood [affective] disorders); F412 * (mixed anxiety and depressive disorder) *
Anxiety disorders	300 (except 3004); 3000 (anxiety states); 3002 (phobic anxiety disorders); 3003 (obsessive-compulsive disorder); 3001 (hysteria); 3006 (other anxiety disorder); 313 (disturbance of emotions specific to childhood and adolescence)	F40 (phobic anxiety disorders); F41(other anxiety disorders); F42 (obsessive-compulsive disorder); F45 (somatoform disorders); F48 (other neurotic disorders); F93, F94 (disturbance of emotions specific to childhood and adolescence)
Adjustment disorders	3090 (brief depressive reaction); 3092 (adjustment reaction with predominant disturbance of other emotions, include: abnormal separation anxiety); 3093 (adjustment reaction with predominant disturbance of conduct); 3094 (adjustment reaction with predominant disturbance of other emotions and conduct); 3098 (other specified adjustment reactions); 3099 (unspecified adjustment reaction)	F430 (acute stress reaction); F431 (post-traumatic stress disorder); F432 (adjustment disorders); F438 (other reactions to severe stress); F439 (reaction to severe stress, unspecified)
Attention deficit/hyperactivity disorder	314 (attention deficit/hyperactivity disorder)	F900; F901; F908; F909 (attention deficit/hyperactivity disorder)
SRDs ^a^		
Alcohol-related disorders	3030 *, 3039 *, 3050 * (alcohol abuse or dependence); 2910 *, 2918 * (alcohol withdrawal), 2911 *–2915 *, 2919 *, 3575, 4255, 5353, 5710-5713 (alcohol-induced disorders); 9800, 9801, 9808, 9809 (alcohol intoxication)	F101 *, F102 * (alcohol abuse or dependence); F103, F104 * (alcohol withdrawal); F105-F109, K700 *–K704 *, K709 *, G621 *, I426, K292 *, K852, K860, E244, G312, G721, O354 (alcohol-induced disorders); F100 *, T510, T511 *, T518, T519 (alcohol intoxication)
Cannabis-related disorders	3043, 3052 (cannabis abuse or dependence)	F121, F122 (cannabis abuse or dependence); F123–F129 (cannabis-induced disorders); F120, T407 (cannabis intoxication)
Drug-related disorders other than cannabis	3040–3042, 3044–3049, 3053–3057, 3059 (drug abuse or dependence); 292.0 (drug withdrawal); 2921, 2922, 2928, 2929 (drug-induced disorders); 9650, 9658, 9670, 9676, 9678, 9679, 9694–9699, 9708, 9820, 9828 (drug intoxication)	F111, F131, F141, F151, F161, F181, F191, F112, F132, F142, F152, F162, F182, F192 (drug abuse or dependence); F113–F114, F133–F134, F143–F144, F153–F154, F163–F164, F183–F184, F193–F194 (drug withdrawal) F115–F119, F135–F139, F145–F149, F155–F159, F165–F169, F185–F189, F195–F199 (drug-induced disorders); F110, F130, F140, F150, F160, F180, F190, T400-T406, T408, T409, T423, T424, T426, T427, T435, T436, T438, T439, T509, T528, T529 (drug intoxication)
Suicide attempts ^b^	E950–E959	X60–X84; Y87.0
Chronic physical illnesses ^a,c^		
Renal failure	4030, 4031, 4039, 4040, 4041, 4049, 585, 586, 5880, V420, V451, V56	I120, I131, N18, N19, N250, Z49, Z940, Z992
Cerebrovascular illnesses	430–438	G45, G46, I60–I69
Neurological illnesses	3319, 3320, 3321, 3334, 3335, 3339, 334–335, 3362, 340, 341, 345, 3481, 3483, 7803, 7843	G10–G12, G13, G20, G21–G22, G254, G255, G312, G318, G319, G32, G35, G36, G37, G40, G41, G931, G934, R470, R56
Endocrine illnesses (hypothyroidism; fluid electrolyte disorders and obesity)	2409, 243, 244, 2461, 2468; 2536, 276; 2780	E00, E01, E02, E03, E890; E222, E86, E87; E66
Any tumor with or without metastasis (solid tumor without metastasis; lymphoma)	140–172, 174, 175, 179–195, 196–199; 200, 201, 202, 2030, 2386, 2733	C00–C26, C30–C34, C37–C41, C43, C45–C58, C60–C76, C77–C79, C80; C81–C85, C88, C900, C902, C96
Chronic pulmonary illnesses	490–505, 5064, 5081, 5088	I278, I279, J40–J47, J60–J64, J65, J66, J67, J684, J701, J703
Diabetes complicated and uncomplicated	2500-2502, 2503; 2504-2509	E102–E108, E112-E118, E132–E138, E142–E148; E100, E101, E109, E110, E111, E119, E130, E131, E139, E140, E141, E149
Cardiovascular illnesses (congestive heart failure; cardiac arrhythmias; valvular illnesses; peripheral vascular illnesses; myocardial infarction; hypertension and pulmonary circulation illnesses)	4021, 4041, 428; 4260, 4267, 4269,4270–4274,4276–4279, 7850, V450, V533; 394–397, 424,7463–7466, V422, V433; 093, 440, 441, 4431–4439, 4471, 5571, 5579, V434; 4109, 4129; 4010, 4011, 4019, 4020, 4021, 4029, 4050, 405,4051, 4059, 4372; 4150, 4151, 416; 4170, 4178, 4179	I099, I110, I130, I132, I255, I420, I425–I429, I43, I50, P290; I441–I443, I456, I459, I47–I49, R000, R001, R008, T821, Z450, Z950; A520, I70–I72, I730, I731, I738, I739, I771, I790, K551, K558, K559, Z958, Z959; I05–I08, I091, I098, I34–I39, Q230–Q233, Q238, Q239, Z952, Z953, Z954, I210–I214, I219, I220, I221, I228, I229, I252; I101, I100, I11, I1500, I1501, I1510, I1511, I1521, I1581, I1590, I1591, I674; I26, I27, I280, I288, I289
Other chronic physical illness categories (blood loss anemia; ulcer illnesses; liver illnesses; AIDS/HIV; rheumatoid arthritis/collagen vascular illnesses, coagulopathy; weight loss, paralysis; deficiency anemia)	2800, 2809; 286, 2871, 2873–2875; 5317, 5319, 5327, 5329, 5337, 5339, 5347, 5349; 0702, 0703, 0704, 0705, 4560–4562, 5723, 5728, 5733, 5734, 5739, V427; 042–044; 1361, 446; 7010, 7100–7104, 7105, 7108, 7109, 7112, 714, 7193, 720, 725, 7285, 7288, 7293; 260–263, 7832, 7994; 3341, 342, 343, 3440–3446, 3448, 3449; 2801, 2809, 281, 2859	D500; K257, K259, K267, K269, K277, K279, K287, K289; B20–B24; D65–D68, D691, D693-D696; B18, I85, I864, I982, K700–K703, K709 K711, K713–K715, K716, K717, K721, K729, K73, K74, K754, K760, K761, K763, K764, K765, K766, K768, K769, Z944; L900, L940, L941, L943, M05, M06, M08, M120, M123, M30, M31, M32–M35, M45, M460, M461, M468, M469; G041, G114, G80, G81, G82, G83; E40–E46, R634, R64, D51–D53, D63, D649; D501, D508; D509
	^a^ All diagnoses identified in RAMQ (*Régie de l’assurance maladie du Québec*, Physician Claims Database) for the full study period were based on the International Classification of Diseases Ninth Revision (ICD-9), which included a 4-digit code, for the financial year 1 April to 31 March. The Canadian Tenth Revision (ICD-10-CA) was used in MED-ECHO (*Maintenance et exploitation des données pour l ’étude de la clientele hospitalières*, Hospital Inpatient and Day Surgery Database) and in the BDCU (*Banque de données communes des urgences*, Emergency Department Use Database). All diagnoses related to the above databases were considered, and all data integrated each year, for each patient. MED-ECHO is the only database that includes several diagnoses: principal diagnosis and numerous secondary diagnoses. In the databases used in this study, MDs were considered only as principal diagnoses, but SRDs as both principal and secondary diagnoses, considering that SRDs are often underdiagnosed. ^b^ Suicide attempts were identified in MED-ECHO (principal and secondary diagnoses), and suicidal behaviors (suicide ideation/attempt) detected in the BDCU by the nurses in ED triage (however not registered in this table as diagnoses, but reasons for ED use). ^c^ The list of chronic physical illnesses is based on an adapted and validated version of the Elixhauser Comorbidity Index, integrating the Charlson Index, which consists of 32 major categories of physical illnesses (see reference in the Methods section). In this list of chronic physical illnesses, three categories of MDs and two of SRDs (identified with an asterisk [*]) were also included in the list of MDs-SRDs, thus appearing twice.	

## References

[1] Barratt H., Rojas-Garcia A., Clarke K., Moore A., Whittington C., Stockton S., Thomas J., Pilling S., Raine R. (2016). Epidemiology of Mental Health Attendances at Emergency Departments: Systematic Review and Meta-Analysis. PLoS ONE.

[2] Santo L., Peters Z.J., De Frances C.J. (2021). Emergency Department Visits among Adults with Mental Health Disorders: United States, 2017–2019.

[3] Fleury M.J., Fortin M., Rochette L., Grenier G., Huỳnh C., Pelletier E., Vasiliadis H.M. (2019). Assessing quality indicators related to mental health emergency room utilization. BMC Emerg. Med..

[4] Fleury M.-J., Fortin M., Rochette L., Lesage A., Vasiliadis H.-M., Huỳn C., Grenier G., Pelletier E. (2019). Surveillance de L’utilisation des Urgences au Québec par Les Patients Ayant des Troubles Mentaux.

[5] Gaulin M., Simard M., Candas B., Lesage A., Sirois C. (2019). Combined impacts of multimorbidity and mental disorders on frequent emergency department visits: A retrospective cohort study in Quebec, Canada. CMAJ.

[6] LaCalle E., Rabin E. (2010). Frequent users of emergency departments: The myths, the data, and the policy implications. Ann. Emerg. Med..

[7] Liu S.W., Nagurney J.T., Chang Y., Parry B.A., Smulowitz P., Atlas S.J. (2013). Frequent ED users: Are most visits for mental health, alcohol, and drug-related complaints?. Am. J. Emerg. Med..

[8] Moe J., Bailey A.L., Oland R., Levesque L., Murray H. (2013). Defining, quantifying, and characterizing adult frequent users of a suburban Canadian emergency department. Can. J. Emerg. Med..

[9] Dixe M.A., Passadouro R., Peralta T., Ferreira C., Lourençco G., Sousa P.M.L. (2018). Determinants of non-urgent emergency department use. Rev. Enferm. Ref..

[10] Hitzek J., Fischer-Rosinsky A., Mockel M., Kuhlmann S.L., Slagman A. (2022). Influence of Weekday and Seasonal Trends on Urgency and In-hospital Mortality of Emergency Department Patients. Front. Public Health.

[11] Kurdyak P., Gandhi S., Holder L., Rashid M., Saunders N., Chiu M., Guttmann A., Vigod S. (2021). Incidence of Access to Ambulatory Mental Health Care Prior to a Psychiatric Emergency Department Visit among Adults in Ontario, 2010–2018. JAMA Netw Open.

[12] Moreno-Kustner B., Warnke I., Nordt C., Fernandez G., Ramos J., Paulino-Matos P., Rossler W., Cardoso G. (2017). Predictors of repeat visits to hospital psychiatric emergency departments in Malaga (Spain) and in Lisbon (Portugal). Emerg. Med. J..

[13] Burns T.R. (2017). Contributing factors of frequent use of the emergency department: A synthesis. Int. Emerg. Nurs..

[14] Minassian A., Vilke G.M., Wilson M.P. (2013). Frequent emergency department visits are more prevalent in psychiatric, alcohol abuse, and dual diagnosis conditions than in chronic viral illnesses such as hepatitis and human immunodeficiency virus. J. Emerg. Med..

[15] Slankamenac K., Heidelberger R., Keller D.I. (2020). Prediction of Recurrent Emergency Department Visits in Patients with Mental Disorders. Front. Psychiatry.

[16] Moe J., Kirkland S., Ospina M.B., Campbell S., Long R., Davidson A., Duke P., Tamura T., Trahan L., Rowe B.H. (2016). Mortality, admission rates and outpatient use among frequent users of emergency departments: A systematic review. Emerg. Med. J. EMJ.

[17] Woo S.E., Jebb A.T., Tay L., Parrigon S. (2018). Putting the “Person” in the Center: Review and Synthesis of Person-Centered Approaches and Methods in Organizational Science. Organ. Res. Methods.

[18] Birmingham L.E., Cochran T., Frey J.A., Stiffler K.A., Wilber S.T. (2017). Emergency department use and barriers to wellness: A survey of emergency department frequent users. BMC Emerg. Med..

[19] Osawa I., Sato T., Goto T., Sonoo T., Iwai S., Nakajima S. (2020). Characteristics and subgroups of frequent emergency department users in an academic hospital in Japan. Acute Med. Surg..

[20] Dufour I., Dubuc N., Chouinard M.C., Chiu Y., Courteau J., Hudon C. (2021). Profiles of Frequent Geriatric Users of Emergency Departments: A Latent Class Analysis. J. Am. Geriatr. Soc..

[21] Moe J., O’Sullivan F., McGregor M.J., Schull M.J., Dong K., Holroyd B.R., Grafstein E., Hohl C.M., Trimble J., McGrail K.M. (2021). Identifying subgroups and risk among frequent emergency department users in British Columbia. J. Am. Coll Emerg. Physicians Open.

[22] Chiu Y.M., Dufour I., Courteau J., Vanasse A., Chouinard M.C., Dubois M.F., Dubuc N., Elazhary N., Hudon C. (2022). Profiles of frequent emergency department users with chronic conditions: A latent class analysis. BMJ Open.

[23] Rinehart D.J., Oronce C., Durfee M.J., Ranby K.W., Batal H.A., Hanratty R., Vogel J., Johnson T.L. (2018). Identifying Subgroups of Adult Superutilizers in an Urban Safety-Net System Using Latent Class Analysis: Implications for Clinical Practice. Med. Care.

[24] Hewlett M.M., Raven M.C., Graham-Squire D., Evans J.L., Cawley C., Kushel M., Kanzaria H.K. (2023). Cluster Analysis of the Highest Users of Medical, Behavioral Health, and Social Services in San Francisco. J. Gen. Intern. Med..

[25] Fleury M.J., Cao Z., Grenier G., Ferland F. (2023). Profiles of quality of life among patients using emergency departments for mental health reasons. Health Qual. Life Outcomes.

[26] Gabet M., Cao Z., Fleury M.J. (2022). Profiles, Correlates and Outcomes Among Patients Experiencing an Onset of Mental Disorder Based on Outpatient Care Received Following Index Emergency Department Visits. Can. J. Psychiatry.

[27] Armoon B., Cao Z., Grenier G., Meng X., Fleury M.J. (2022). Profiles of high emergency department users with mental disorders. Am. J. Emerg. Med..

[28] Moulin A., Evans E.J., Xing G., Melnikow J. (2018). Substance Use, Homelessness, Mental Illness and Medicaid Coverage: A Set-Up for High Emergency Department Utilization. Health Qual. Life Outcomes.

[29] Fleury M.J., Cao Z., Grenier G., Huynh C. (2023). Predictors of Death From Physical Illness or Accidental/Intentional Causes among Patients with Substance-Related Disorders. Can. J. Psychiatry.

[30] Gale C.R., Batty G.D., Osborn D.P., Tynelius P., Whitley E., Rasmussen F. (2012). Association of mental disorders in early adulthood and later psychiatric hospital admissions and mortality in a cohort study of more than 1 million men. Arch. Gen. Psychiatry.

[31] Vandenbroucke J.P., von Elm E., Altman D.G., Gotzsche P.C., Mulrow C.D., Pocock S.J., Poole C., Schlesselman J.J., Egger M., Strobe Initiative (2007). Strengthening the Reporting of Observational Studies in Epidemiology (STROBE): Explanation and elaboration. Epidemiology.

[32] Billings J., Raven M.C. (2013). Dispelling an urban legend: Frequent emergency department users have substantial burden of disease. Health Aff. (Millwood).

[33] Canadian Association of Emergency Physicians Canadian Triage Acuity Scale. https://caep.ca/wp-content/uploads/2017/06/module_I_slides_v2.5_2012.pdf.

[34] Moe J., Wang Y.E., Schull M.J., Dong K., McGregor M.J., Hohl C.M., Holroyd B.R., McGrail K.M. (2022). Characterizing people with frequent emergency department visits and substance use: A retrospective cohort study of linked administrative data in Ontario, Alberta, and B.C., Canada. BMC Emerg. Med..

[35] Pampalon R., Hamel D., Gamache P., Raymond G. (2009). A deprivation index for health planning in Canada. Chronic Dis. Can..

[36] Simard M., Sirois C., Candas B. (2018). Validation of the Combined Comorbidity Index of Charlson and Elixhauser to Predict 30-Day Mortality across ICD-9 and ICD-10. Med. Care.

[37] Deyo R.A., Cherkin D.C., Ciol M.A. (1992). Adapting a clinical comordibity index for use with ICD-9-CM Administrative Databases. J. Clin. Epidemiol..

[38] Carter R., Quesnel-Vallee A., Plante C., Gamache P., Levesque J.F. (2016). Effect of family medicine groups on visits to the emergency department among diabetic patients in Quebec between 2000 and 2011: A population-based segmented regression analysis. BMC Fam. Pract..

[39] Kappelin C., Carlsson A.C., Wachtler C. (2022). Specific content for collaborative care: A systematic review of collaborative care interventions for patients with multimorbidity involving depression and/or anxiety in primary care. Fam. Pract..

[40] Breslau N., Reeb K.G. (1975). Continuity of care in a university-based practice. J. Med. Educ..

[41] Ionescu-Ittu R., McCusker J., Ciampi A., Vadeboncoeur A.M., Roberge D., Larouche D., Verdon J., Pineault R. (2007). Continuity of primary care and emergency department utilization among elderly people. CMAJ.

[42] Wang P.S., Demler O., Kessler R.C. (2002). Adequacy of Treatment for Serious Mental Illness in the United States. Am. J. Public Health.

[43] Young A.S., Klap R., Shoai R., Wells K.B. (2008). Persistent depression and anxiety in the United States: Prevalence and quality of care. Psychiatr. Serv..

[44] Fleury M.J., Grenier G., Vallee C., Aube D., Farand L., Bamvita J.M., Cyr G. (2016). Implementation of the Quebec mental health reform (2005–2015). BMC Health Serv. Res..

[45] Moorin R.E., Youens D., Preen D.B., Wright C.M. (2020). The association between general practitioner regularity of care and ‘high use’ hospitalisation. BMC Health Serv. Res..

[46] Thibodeau L., Rahme E., Lachaud J., Pelletier E., Rochette L., John A., Reneflot A., Lloyd K., Lesage A. (2018). Individual, programmatic and systemic indicators of the quality of mental health care using a large health administrative database: An avenue for preventing suicide mortality. Health Promot. Chronic Dis. Prev. Can..

[47] Hermann R.C., Mattke S., Somekh D., Silfverhielm H., Goldner E., Glover G., Pirkis J., Mainz J., Chan J.A. (2006). Quality indicators for international benchmarking of mental health care. Int. J. Qual. Health Care.

[48] Samartzis L., Talias M.A. (2019). Assessing and Improving the Quality in Mental Health Services. Int. J. Environ. Res. Public Health.

[49] Bartlett J.W., Harel O., Carpenter J.R. (2015). Asymptotically Unbiased Estimation of Exposure Odds Ratios in Complete Records Logistic Regression. Am. J. Epidemiol..

[50] Goodman L.A. (1974). Exploratory latent structure analysis using both identifiable and unidentifiable models. Biometrika.

[51] Lazarsfeld P.F., Henry N.W. (1968). Latent Structure Analysis.

[52] Hagernaars J.A., McCutcheon A.L. (2009). Applied Latent Class Analysis.

[53] Schwarz G. (1978). Estimating the dimension of a model. Ann. Stat..

[54] Celeux G., Soromenho G. (1996). An entropy criterion for assessing the number of clusters in a mixture model. J. Classif..

[55] Lanza S.T., Collins L.M., Lemmon D.R., Schafer J.L. (2007). PROC LCA: A SAS Procedure for Latent Class Analysis. Struct. Equ. Model..

[56] StataCorp (2021). Stata Statistical Software 17.

[57] Chang G., Weiss A.P., Orav E.J., Rauch S.L. (2014). Predictors of frequent emergency department use among patients with psychiatric illness. Gen. Hosp. Psychiatry.

[58] Vu F., Daeppen J.B., Hugli O., Iglesias K., Stucki S., Paroz S., Canepa Allen M., Bodenmann P. (2015). Screening of mental health and substance users in frequent users of a general Swiss emergency department. BMC Emerg. Med..

[59] Fleury M.J., Rochette L., Grenier G., Huynh C., Vasiliadis H.M., Pelletier E., Lesage A. (2019). Factors associated with emergency department use for mental health reasons among low, moderate and high users. Gen. Hosp. Psychiatry.

[60] Richard-Lepouriel H., Weber K., Baertschi M., DiGiorgio S., Sarasin F., Canuto A. (2015). Predictors of recurrent use of psychiatric emergency services. Psychiatr. Serv..

[61] Moller-Leimkuhler A.M. (2002). Barriers to help-seeking by men: A review of sociocultural and clinical literature with particular reference to depression. J. Affect. Disord..

[62] Urbanoski K., Inglis D., Veldhuizen S. (2017). Service Use and Unmet Needs for Substance Use and Mental Disorders in Canada. Can. J. Psychiatry.

[63] Saillant S., Hudelson P., Dominice Dao M., Junod Perron N. (2016). The primary care physician/psychiatrist joint consultation: A paradigm shift in caring for patients with mental health problems?. Patient Educ. Couns..

[64] Davy C., Bleasel J., Liu H., Tchan M., Ponniah S., Brown A. (2015). Effectiveness of chronic care models: Opportunities for improving healthcare practice and health outcomes: A systematic review. BMC Health Serv. Res..

[65] Fleury M.J., Grenier G., Gentil L., Roberge P. (2021). Deployment of the consultation-liaison model in adult and child-adolescent psychiatry and its impact on improving mental health treatment. BMC Fam. Pract..

[66] Coventry P.A., Hudson J.L., Kontopantelis E., Archer J., Richards D.A., Gilbody S., Lovell K., Dickens C., Gask L., Waheed W. (2014). Characteristics of effective collaborative care for treatment of depression: A systematic review and meta-regression of 74 randomised controlled trials. PLoS ONE.

[67] Bost N., Crilly J., Wallen K. (2015). The impact of a flow strategy for patients who presented to an Australian emergency department with a mental health illness. Int. Emerg. Nurs..

[68] Johnston A.N., Spencer M., Wallis M., Kinner S.A., Broadbent M., Young J.T., Heffernan E., Fitzgerald G., Bosley E., Keijzers G. (2019). Review article: Interventions for people presenting to emergency departments with a mental health problem: A systematic scoping review. Emerg. Med. Australas..

[69] Krieg C., Hudon C., Chouinard M.C., Dufour I. (2016). Individual predictors of frequent emergency department use: A scoping review. BMC Health Serv. Res..

[70] Gentil L., Grenier G., Vasiliadis H.M., Huynh C., Fleury M.J. (2021). Predictors of Recurrent High Emergency Department Use among Patients with Mental Disorders. Int. J. Environ. Res. Public Health.

[71] Fahimi J., Aurrecoechea A., Anderson E., Herring A., Alter H. (2015). Substance abuse and mental health visits among adolescents presenting to US emergency departments. Pediatr. Emerg. Care.

[72] Pines J.M., Asplin B.R., Kaji A.H., Lowe R.A., Magid D.J., Raven M., Weber E.J., Yealy D.M. (2011). Frequent users of emergency department services: Gaps in knowledge and a proposed research agenda. Acad. Emerg. Med..

[73] Gentil L., Grenier G., Meng X., Fleury M.J. (2021). Impact of Co-occurring Mental Disorders and Chronic Physical Illnesses on Frequency of Emergency Department Use and Hospitalization for Mental Health Reasons. Front. Psychiatry.

[74] Castillejos Anguiano M.C., Martin Perez C., Bordallo Aragon A., Sepulveda Manoz J., Moreno Kiustner B. (2020). Patterns of primary care among persons with schizophrenia: The role of patients, general practitioners and centre factors. Int. J. Ment. Health Syst..

[75] Gentil L., Grenier G., Vasiliadis H.M., Fleury M.J. (2022). Predictors of Length of Hospitalization and Impact on Early Readmission for Mental Disorders. Int. J. Environ. Res. Public Health.

[76] Fleury M.J., Cao Z., Armoon B., Grenier G., Lesage A. (2022). Profiles of patients using emergency departments or hospitalized for suicidal behaviors. Suicide Life-Threat. Behav..

[77] Mueser K.T., Noordsy D.L., Drake R.E., Fox L. (2003). Integrated Treatment for Dual Disorders: A Guide to Effective Practice.

[78] Zhang S., Mellsop G., Brink J., Wang X. (2015). Involuntary admission and treatment of patients with mental disorder. Neurosci. Bull..

[79] Dieterich M., Irving C.B., Park B., Marshall M. (2017). Intensive case management for severe mental illness. Cochrane Database Syst. Rev..

[80] Penzenstadler L., Soares C., Anci E., Molodynski A., Khazaal Y. (2019). Effect of Assertive Community Treatment for Patients with Substance Use Disorder: A Systematic Review. Eur. Addict. Res..

[81] Gabet M., Armoon B., Meng X., Fleury M.J. (2023). Effectiveness of emergency department based interventions for frequent users with mental health issues: A systematic review. Am. J. Emerg. Med..

[82] Loeb D.F., Bayliss E.A., Binswanger I.A., Candrian C., deGruy F.V. (2012). Primary care physician perceptions on caring for complex patients with medical and mental illness. J. Gen. Intern. Med..

[83] Siskind D., Harris M., Kisely S., Brogan J., Pirkis J., Crompton D., Whiteford H. (2013). A retrospective quasi-experimental study of a community crisis house for patients with severe and persistent mental illness. Aust. N. Z. J. Psychiatry.

[84] Gabet M., Grenier G., Cao Z., Fleury M.J. (2020). Implementation of three innovative interventions in a psychiatric emergency department aimed at improving service use: A mixed-method study. BMC Health Serv. Res..

[85] Anderson K., Goldsmith L.P., Lomani J., Ali Z., Clarke G., Crowe C., Jarman H., Johnson S., McDaid D., Pariza P. (2022). Short-stay crisis units for mental health patients on crisis care pathways: Systematic review and meta-analysis. BJPsych Open.

[86] Lamb D., Lloyd-Evans B., Fullarton K., Kelly K., Goater N., Mason O., Gray R., Osborn D., Nolan F., Pilling S. (2020). Crisis resolution and home treatment in the UK: A survey of model fidelity using a novel review methodology. Int. J. Ment. Health Nurs..

[87] Fleury M.J., Imboua A., Grenier G. (2024). Barriers and Facilitators to High Emergency Department Use Among Patients with Mental Disorders: A Qualitative Investigation. Community Ment. Health J..

[88] Fleury M.J., Perreault M., Grenier G., Imboua A., Brochu S. (2016). Implementing Key Strategies for Successful Network Integration in the Quebec Substance-Use Disorders Programme. Int. J. Integr. Care.

